# The potential role of collagen type VII in breast cancer proliferation

**DOI:** 10.1186/s12935-024-03449-4

**Published:** 2024-07-20

**Authors:** Sergio Pérez-Díaz, Jessica Lindberg, Luis Oliveros Anerillas, Paul J. Kingham, Malin Sund, Gunilla Rask, Johan Svensson, Malin Jansson, Rebecca Wiberg

**Affiliations:** 1 Department of Medical and Translational Biology, Umeå University, Umeå, SE-901 87 Sweden; 2 Department of Diagnostics and Intervention, Plastic Surgery and Surgery, Umeå University, Umeå, Sweden; 3 Department of Surgery/CLINICUM, University of Helsinki and Helsinki University Hospital, Helsinki, Finland; 4 Department of Statistics, Umeå School of Business, Economics and Statistics, Umeå University, Umeå, Sweden; 5 Present Address: Department of Laboratory Medicine, Division of Clinical Physiology, Karolinska Institute, Stockholm, Sweden

**Keywords:** Breast cancer, Collagen type VII, Extracellular matrix, Mesenchymal stem cell

## Abstract

**Background:**

Breast cancer is the most common cancer in women. Cancer cells can persist in a prolonged dormant state for years without any clinical evidence of disease creating an urgent need to better understand the molecular mechanisms leading to relapse. This study aimed to identify extracellular matrix (ECM) components associated with hypoxia-induced breast cancer dormancy. The effects of selected ECM proteins on breast cancer cell proliferation were analyzed, along with their correlation with established prognostic markers in human breast cancer tissue.

**Materials and methods:**

Screening of extracellular matrix proteins was performed in hypoxia-induced dormant MCF-7 breast cancer cells. Proliferation of MCF-7 cells in vitro was subsequently determined in the presence of recombinant ColVII. Adipose tissue-derived mesenchymal stem cells (AdMSCs) subpopulation overexpressing ColVII were indirectly isolated by ColVII receptor integrin-α6 specific antibodies. AdMSCs- MCF-7 3D spheroid cultures were generated to model solid tumour conditions. In addition, the association between ColVII and various prognostic markers was evaluated in clinical samples of human breast cancer tissue.

**Results:**

Dormant MCF-7 cells showed an elevated expression of ColVII while MCF-7 cells cultured on ColVII exhibited reduced proliferation in vitro. In AdMSCs-MCF-7 3D spheroids, a reduced proliferation of MCF-7 cells was observed in Int-α6^+^/ ColVII_high_ compared with Int-α6^-/^ ColVII_low_ AdMSCs spheroids. In human tissue, high ColVII expression correlated to several positive prognostic markers. Staining for Cytokeratin-5 revealed that ColVII_high_-expressing cells were predominantly myoepithelial cells.

**Conclusion:**

ColVII is associated with reduced proliferation of breast cancer cells in vitro. ColVII is strongly expressed in myoepithelial cells and in breast cancer tissue the high ColVII expression correlates with several well-known positive prognostic markers, highlighting its potential as a prognostic marker in breast cancer.

**Supplementary Information:**

The online version contains supplementary material available at 10.1186/s12935-024-03449-4.

## Introduction

Breast cancer is the most common cancer worldwide accounting for more than 2 million women diagnosed with breast cancer in 2020 [1]. Breast cancer cells are capable of surviving in a prolonged dormant, non-proliferative state for years without any clinical evidence of disease after definitive treatment of the primary tumour [2]. Due to a decreased proliferation rate, quiescent- dormant cancer cells are unresponsive to classical chemotherapy treatments directed against cells with a rapid cell cycle [3]. In addition, dormant cancer cells can become reactivated, disseminate, and emerge as a relapse [4]. A large part of cancer-related deaths in women is caused by such metastasized breast cancer [5]. Thus, there is an urgent need to better understand the molecular mechanisms leading to relapse after treatment.

The mammary gland is surrounded by a basement membrane and a connective tissue stroma, composed of extracellular matrix (ECM) and heterogenous cell populations and tissues including endothelial cells, immune cells, fibroblasts and the abundant breast adipose tissue with its associated mesenchymal stem/ progenitor cells (AdMSCs) [6]. Due to the intimate localization of the mammary glands and the breast adipose tissue, there is a close crosstalk between breast cancer cells and the AdMSCs [7]. Recent studies have shown that AdMSCs exposed to breast cancer cells-conditioned media lose multipotency and increase the expression of myofibroblast markers and various ECM factors [8, 9]. The ECM, a structural meshwork of different components including glycosaminoglycans, proteoglycans, laminins, collagens, and remodeling enzymes, is increasingly recognized as an important regulator in breast cancer. The ECM shows numerous changes in composition and organization in breast cancer development when compared with the mammary gland under homeostasis [10]. Changes in stiffness and composition in fibrillar collagens such as type I, III, and V collagen and non-fibrillar like type IV collagen (ColIV) has been observed at different breast cancer stages [10–12].

ColVII, a non-fibrillar member of the collagen family, is very abundant in the skin where it is mainly produced by keratinocytes and fibroblasts and is the major component of anchoring fibrils in the basement membrane [13] that anchors the epidermis to dermis. The role of ColVII has been extensively studied in recessive dystrophic epidermolysis bullosa, where mutation in *COL7A1*, the gene encoding ColVII, mediates extensive blistering of the skin [14]. Recent studies have indicated a protective role of ColVII in breast cancer [15], however the mechanisms behind ColVII in cancer progression are poorly understood.

The first aim of this study was to unveil ECM components associated to hypoxia-induced breast cancer dormancy. The screen identified that ColVII expression was markedly up-regulated so the following studies analyzed the role of ColVII in breast cancer cell´s proliferation and investigated the association between ColVII and other known prognostic markers in human breast cancer tissue.

## Materials and methods

### Cell cultures and treatments

Human adipose tissue samples were obtained from discarded material after liposuction at Umeå University Hospital, Sweden (*n* = 2 donors). Aspirated samples were treated as previously described to yield stromal vascular fraction cell pellets containing the AdMSCs [16]. Once the cultures were established, donor lines were pooled. Non-tumorigenic MCF-12 human epithelial breast cells were purchased from ATCC (#CRL-3598, Merck Life Sciences, Darmstadt, Germany). Human estrogen-positive breast cancer MCF-7 (ATCC, #HTB-22, Merck Life Sciences) cells expressing green fluorescent protein (GFP) were kindly donated by Iwan Jones (Section of Molecular Medicine, Umeå University, Umeå, Sweden). The cultures were expanded and subsequently maintained in vitro using Dulbecco′s Modified Eagle′s Medium low glucose (DMEM, ThermoFisher, Gibco^®^, Waltham, MA, USA, #11,885,084) containing 10% (v/v) FBS (Sigma-Aldrich. #F9665) and 1% (v/v) penicillin/streptomycin (ThermoFisher. Gibco^®^, #15,140,122) and incubated at 37 °C with 5% CO_2_ in T75 flasks. Cultures at 80–90% confluence were washed with Dulbecco’s Phosphate Buffered Saline (DPBS, (ThermoFisher, Gibco^®^, #14,190,144), detached using TrypLE (ThermoFisher. Gibco^®^ #12,563,029) and cryopreserved in 90% (v/v) FBS (Sigma-Aldrich #F9665) with 10% (v/v) dimethyl sulfoxide (Sigma-Aldrich, St. Louis, MO, USA, #D2650) and stored in liquid nitrogen. Cells were counted and seeded according to the experimental setting or the supernatants collected and filtered with 0.2 μm filter and used as conditioned media. To induce dormancy, a previously described hypoxic dormancy model using Cobalt chloride (CoCl_2_) (Merck Life Sciences, Solna, Sweden) was followed [17]. Cell cultures at 40–50% confluence were treated with 300 µM CoCl_2_ for 5 days. The dormant cells were then allowed either to recover in normal growth media for 3 days, referred to as restored normoxia, or treated with CoCl_2_ for another 3 days, referred to as hypoxia. Culture plates were coated with 2–20 ng/mL human recombinant ColVII (rhColVII; Origene, Rockville, MD, USA, #TP318470) in 0.5% Gelatin (Merck. #G6650) diluted in DPBS for 2 h at 37 °C. Later, the coating solutions were discarded, and the MCF-7 cells seeded according to the proliferation assay. MCF-7 invasiveness and AdMSCs recruitment were evaluated using a migration assay (see below). AdMSCs and MCF-7 were exposed to MCF-7 conditioned media and 2–20 ng/mL of rhColVII. MCF-7 cell growth medium without exposure to the cells and rhColVII-free conditions were used as respective controls.

### Cell sorting

AdMSCs subpopulations with high and low ColVII expression were magnetically sorted (MACS^®^) using an APC conjugated antibody against the ColVII receptor integrin-α6 [18] (Biolegend, San Diego, CA, USA, #313,615) and anti-APC magnetic beads (Miltenyi Biotec, Bergisch Gladbach, Germany #130-090-855).

### Proliferation and migration assays

MCF-7-GFP cells at a density of 5000 cells/cm^2^ were plated in 96-well plates coated with 0-20ng/mL rhColVII in 150 µL of growth medium. Proliferation was indirectly measured by the GFP signal at 488/510nm every 48 h until day 10 with a Synergy HT Microplate Reader (BioTek, Winooski, VT, USA). To assess MCF-7 invasiveness and AdMSCs chemoattraction to the MCF-7´secretome, 30.000 cells were plated in the migration kit trans-well (Cell Biolabs, San Diego, CA, USA, #CBA-106) with 150 µL in serum free media. The trans-wells containing the cells were inserted into the lower compartment containing the different concentrations of soluble rhColVII and MCF-7 conditioned media, respectively. Cell migration through the insert to the lower compartment was measured after 24 h according to the manufacturer’s protocol.

### 3D models and cryosections

To generate AdMSCs-MCF-7 3D spheroids, 200.000 Int-6α + and Int-6α- AdMSCs were transferred into a round-bottom test tube (ThermoFisher, #10,314,791) with 1mL growth medium and centrifuged at 400 g for 5 min. The resulting pellet was incubated at 37 °C, 5% CO_2_, and humid conditions. After 48 h, the tubes containing spherical pellets were supplemented with 25.000 MCF-7-GFP in 500 µL of growth medium. The following day, the supernatants were discarded, and fresh growth medium added to the spheroids until the GFP signal was measured and the histological characterization was performed at days 1 and 5. The GFP signal was measured using an Olympus IX71 microscope (Olympus life science). Spheroids were fixed with 4% (w/v) paraformaldehyde (PFA) (ThermoFisher, #11,586,711) for 2 h at 4 °C, the PFA was removed, and the spheroids washed with DPBS and treated with 30% sucrose overnight. The following day the spheroids were embedded into OCT and frozen in isopentane cooled with liquid nitrogen (VWR, #24872.298) and stored at -80 °C until sectioning (7-µm thick slices) was performed using a CryoStar™ NX50 Cryostat (ThermoFisher).

### Protein analysis

#### Western blot

MCF-7 cells, MCF-12 cells and AdMSCs were treated following the different experimental set-ups and Western blotting performed using standard protocols. The primary and secondary antibodies used are presented in Supplementary Table 1. Chemiluminescence signal was detected in a LI-COR Odyssey^®^ XF using Image Studio Lite Ver. 5.2(LI-COR Biosciences, Lincoln, NE, USA) and the bands quantified with image J (ImageJ software). Western blots against β-Actin were used as loading control to normalize target protein quantifications.

#### Immunocytochemistry

MCF-7 cells, MCF-12 cells, and AdMSCs were plated in 8-well chamber slides (Sarstedt, Nümbrecht, Germany, #94.6140.802) at a density of 2500 cells/cm^2^ in triplicates and treated following the different experimental set-ups. The cultures were fixed in 4% (w/v) PFA and then stained with primary and secondary antibodies shown in Supplementary Table 1. The slides were viewed under an ECLIPSE 90i fluorescence microscope (Nikon Europe BV, Amstelveen, The Netherlands) and images captured with Nikon Elements Imaging software. Cells positive for the expression of Ki67 and α-Smooth Muscle Actin were counted manually. The signal for ColVII, GFP, and Nidogen-1 were measured in pixels per area using a macro for Image J program (developed and kindly donated by Benedicte Chazaud, Institute NeuroMyoGène, Lyon, France). Nuclei staining with 4′,6-diamidino-2-phenylindole (DAPI) was used to quantify the total number of cells in each captured area and calculate the percentage of positive cells, as well as for normalizing the signal values obtained from the above-described macro.

### Gene expression analysis

MCF-7 cells and AdMSCs were seeded in 6-well plates and treated with CoCl_2_ according to the experimental design. The supernatants were removed, and the cultures washed twice with DPBS. Total RNA was isolated using RNeasy mini kits according to the manufacturer’s instructions and up to 500 ng of RNA were retro-transcribed into cDNA with iScript cDNA synthesis kit (BIO-RAD, Hercules, CA, USA, #1,708,891) following the manufacturer’s protocol. The target genes were amplified using specific primers with SsoFast™EvaGreen^®^ supermix (BIO-RAD #1,725,204) in a CFX96 Optical Cycler and analysed using the CFX96 manager software (BIO-RAD). Primers sequences are shown in Supplemental Table 1. Gene expression values were calculated by utilizing the ΔΔCT method (Livak & Schmittgen, 2001) and expressed as the relative change to β-actin and RPL13a.

### Histological analysis of human breast cancer tissue

Tumour tissue samples were collected from 15 female breast cancer patients (7 with estrogen receptor (ER) positive breast cancer and 8 with triple negative breast cancer (TNBC)) undergoing surgery at Umeå University Hospital between 2008 and 2009. The tissue samples were fixed in formalin and embedded as paraffin blocks. Routine staining with haematoxylin and eosin (H&E) was performed using standard protocols. Standard clinicopathological variables including histological grade I-III, hormonal receptor status, *HER2* gene amplification, proliferation marker Ki67, and nodal metastases were analysed by a specialized breast pathologists at the Department of Pathology, Umeå University Hospital following validated local/national guidelines. The immunohistochemical staining of the breast cancer tissue was performed with a Ventana Benchmark automated immunostainer (Ventana Medical Systems, Roche Tissue Diagnostics, Tucson, AZ, USA). Before the protocol was started, the tissue was pre-treated with EDTA. The primary antibody against ColVII (1:100 dilution; polyclonal rabbit, HPA042420 Sigma-Aldrich) was incubated for 44 min at 36 °C followed by an anti-rabbit HRP-conjugated secondary antibody. Thereafter, UV diaminobenzidine tetrahydrochloride (DAB) H_2_O_2_ and UV Copper as chromogen was added and incubated for 8 and 4 min respectively in 36 °C. Finally, haematoxylin II was applied and incubated for 8 min to give contrast. Skin tissue was used as a positive control and liver tissue as a negative control (Supp. Figure 1). The slides were scanned (P250; 3DHistech) into MRXS-files, which were then analysed with the open source bioimaging program Qu Path version 0.4.3 [19]. The image type was set as Brightfield (H-DAB), and colour deconvolution was set using stain vectors to maximize colour differentiation. The settings were saved in a script and applied identically on all samples. An antibody against the myoepithelial cell marker cytokeratin 5 (CK5) was used to stain all samples following validated protocols used in clinical practice at the Department of Pathology, Umeå University Hospital. The tumour areas were marked under the supervision of a breast pathologist and a grid measuring 1000 × 1000 μm was applied on the image and divided into subareas of 106 µm^2^ for further quantification of the staining. A subarea was marked as ColVII positive if any ColVII staining was detected within that subarea. The quantification of ColVII was performed by detecting or not detecting staining in any of the subareas of 106 µm^2^ (present staining/no present staining), and then dividing the number of positive subareas with the previously marked total tumour area, giving a gross percentage of the overall positive staining.

**Fig. 1:**
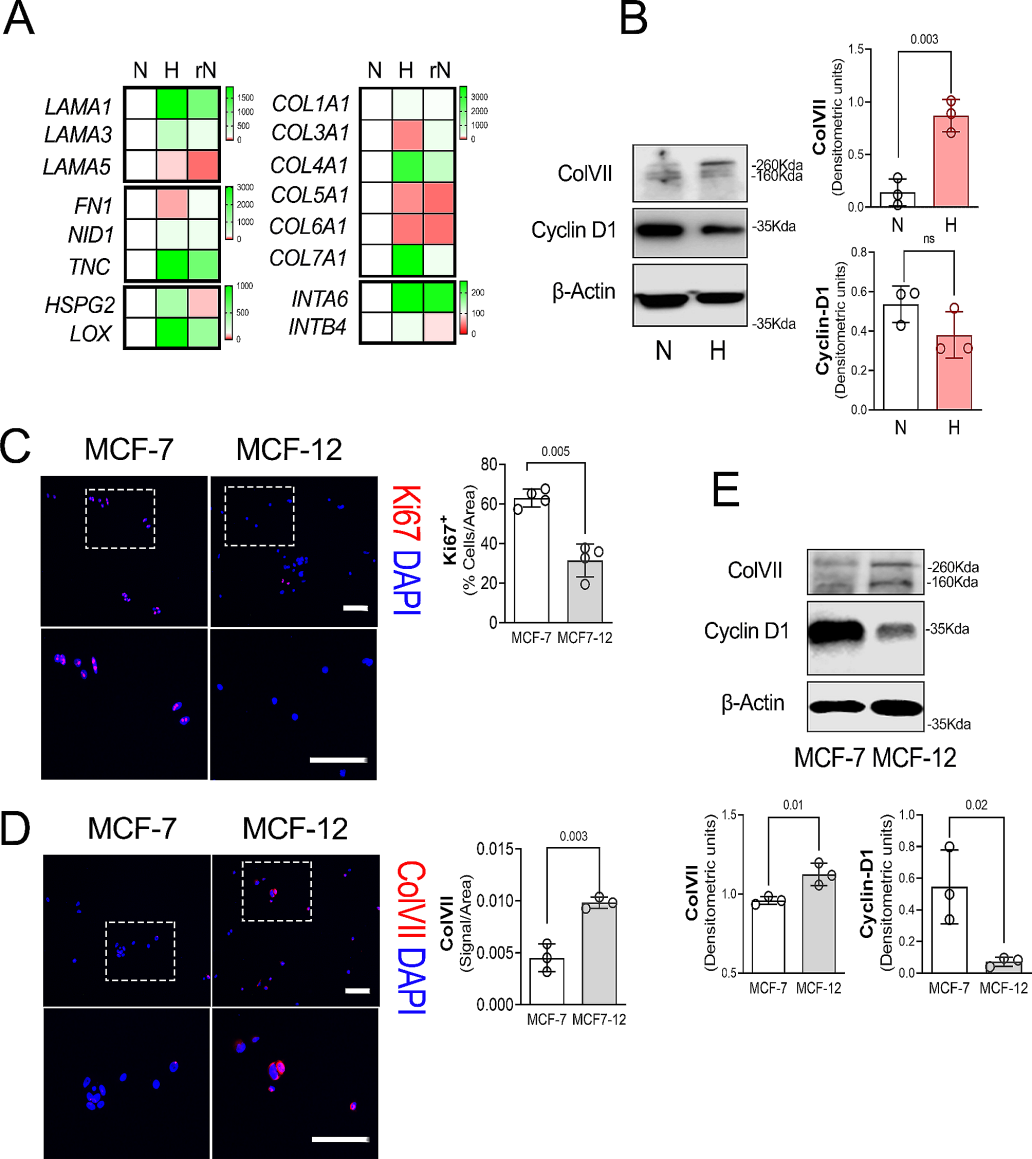
Non-tumourigenic MCF-12 cells express higher Collagen type VII compared with tumourogenic MCF-7 breast cancer line. **A.** Gene expression analysis of laminins; *LAMA1*, *LAMA3*, *LAMA5*, non-collagens; Fibronectin (*FN1*), Nidogen-1 (*NID-1*), Tenascin-C (*TNC*), collagens; *COL1A1*, *COL3A1*, *COL4A1*, *COL5A1*, *COL6A1*, *COL7A1* and integrins (*INTA6* and *INTB4*) in MCF-7 cells exposed to normoxia (N), hypoxia (H) or restored normoxia (rN). Data are expressed as means of triplicates and represent fold change relative to N (100) with colour coding (White = 100, Green > 100 and Red < 100). **B.** Left: Representative western blot against Collagen type VII (ColVII), Cyclin-D1 and β-Actin in MCF-7 cells exposed to normoxia (N) and hypoxia (H). Right: Quantification of Western blot bands (Densitometric units) showing relative expression of the target proteins to β-Actin. Data are expressed as means ± SEM of triplicates. *P*-values represent the t-tests N vs. H. **C.** Left: Representative Ki67 (Red) staining of MCF-7 and MCF-12 cultures. Nuclei are stained with DAPI (blue). Scale bar = 50 μm. Insets: Dotted areas´ magnifications. Right: Quantification in percentage of Ki67 positive MCF-7 and MCF-12 per total cells/area after 5 days of culture. Data are expressed as means ± SEM of four experiments. *P*-values represent the t-tests MCF-7 vs. MCF-12.. **D.** Left: Representative Collagen type VII (Red) staining of MCF-7 and MCF-12 cultures. Nuclei are stained with DAPI (blue). Scale bar = 50 μm. Insets: Dotted areas´ magnifications. Right: Collagen-VII signal quantification (Pixels) in MCF-7 and MCF-12 cultures per area after 5 days of culture. Data are expressed as means ± SEM of triplicates. *P*-values represent the t-tests between MCF-7 vs. MCF-12. **E.** Above: Representative western blot against Collagen type VII (ColVII), Cyclin-D1 and β-Actin in MCF-7 and MCF-12 cells after 5 days of culture. Below: Quantification of Western blot bands (Densitometric units) showing relative expression of the target proteins to β-Actin. Data are expressed as means ± SEM of triplicates. *P*-values represent t-tests MCF-7 vs. MCF-12

### Statistical analysis

In the experimental part, data is expressed as means ± standard error of the mean (SEM) of at least two independent experiments. Statistical differences were determined by paired t-tests when the mean of three independent experiments were compared, and unpaired t-tests when the gross values of the experiments (*n* = 2–3) were compared. Ordinary one-way ANOVA was used when three or more groups were compared. Empirical rule µ ± 1.5 σ score analysis was used to discard outliers. The calculations were performed using GraphPad Prism 8 software (GraphPad Software, San Diego, CA, USA). For the human breast cancer specimens, the correlation between breast cancer subtype and ColVII expression in the tumour area was performed by Mann-Whitney U test. Further, ColVII expression in the tumour area was divided into two groups, one including negative ColVII expression in the tumour area (= 0%) and the other including positive ColVII expression in tumour area (> 0%), and these groups were correlated to different clinicopathological variables by crosstabulation and Fisher’s exact test. The calculations were performed using SPSS Statistic Software (SPSS, Chicago, IL, USA, Version 29.0.1.0 (171)).

## Results

### Hypoxia induced dormancy in MCF-7 cells

To mimic breast cancer cell dormancy, we have simulated hypoxia-induced-dormancy [20] in our MCF-7-GFP breast cancer line with CoCl_2_, a pharmaceutical hypoxia-mimetic agent [17] (Supp. Figure 2A). The hypoxia-induced dormant cells (H) showed lower proliferation as measured by lower RNA content (Supp. Figure 2B), decreased area occupied by GFP^+^ cells (Supp. Figure 2C) and lower expression of the proliferation marker *Ki67* gene (Supp. Figure 2D) when compared with the untreated cells (Normoxia (N)). The cyclin-dependent kinase inhibitor p21, which promotes cell cycle arrest, was increased in the hypoxia-induced dormant cells along with the stem cell markers *NANOG* and *OCT3/4* (Supp. Figure 2D). A hallmark of cellular dormancy is the ability to re-awake upon removal of the environmental stressor that led them to enter dormancy, defined as reversible dormancy [21]. Upon removal of the hypoxic stress, the aforementioned changes were reversible, observed as a restored proliferative capacity and decreased *p21* gene expression in the restored normoxia group (rN) (Supp. Figure 2B-D).

**Fig. 2:**
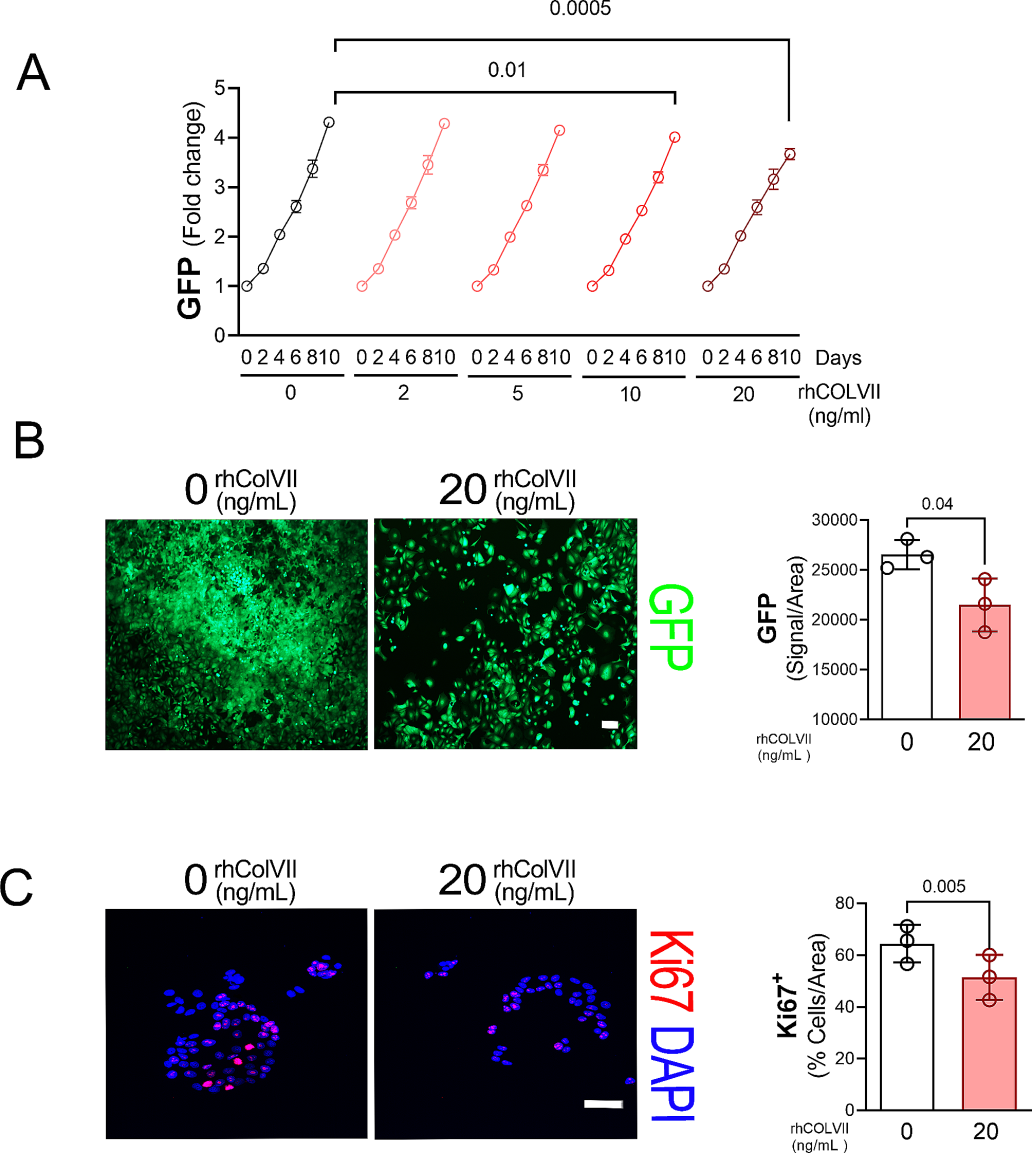
Increased Collagen type VII expression correlates with lower MCF-7 proliferation. **A.** GFP fluorescent signal quantification (488/510 nm) of MCF-7-GFP cells cultured on coated plates with 0, 2, 5, 10 and 20ng/mL rhCol-VII on day 0, 2, 4, 6, 8 and 10. Graphs represent mean ± SEM of triplicates. *P*-values were calculated by t-tests between 0 and 10 ng/mL and between 0 and 20 ng/mL rhColVII on day 10. **B.** Left: Representative GFP images of MCF-7-GFP after 10 days of culture in the presence of 0 and 20 ng/mL of rhCol-VII. Scale bar = 200 μm. Right: Relative GFP signal quantification (Pixels) in MCF-7-GFP after 10 days of culture in presence of 0 and 20 ng/mL of rhColVII. Data are expressed as means ± SEM of triplicates. *P*-values were calculated by t-tests between 0 vs. 20 ng/mL of rhColVII. **C.** Left: Representative immunocytochemical Ki67 (Red) staining of MCF-7 after 10 days of culture in the presence of 0 and 20 ng/mL of rhColVII. Nuclei are stained with DAPI (blue). Scale bar = 50 μm. Right: Ki67 positive MCF-7 quantification per total number of cells after 10 days of culture in presence of 0 and 20 ng/mL of rhColVII. Data are expressed as means ± SEM of triplicates. *P*-values were calculated by t-tests between 0 vs. 20 ng/mL of rhColVII

To investigate the microenvironment of dormant MCF-7 cells, we screened for different ECM molecules by qRT-PCR (Fig. 1A). Laminins, *LAMA1* and *LAMA3*; the glycoprotein Tenascin-C (*TNC*) and the inter-linker *LOX* transcripts were elevated and the *LAMA5* expression downregulated in the hypoxic group compared with the normoxic and restored normoxia groups. The collagen expression for *COL3A1*, *COL5A1* and *COL6A1* were downregulated while *COL4A1* and *COL7A1* were upregulated in the hypoxic group compared with the normoxic and restored normoxia groups. The hypoxic conditions induced the elevated expression of cell-ECM integrin receptors *INTα6* and *INTβ4*. Among the ECM components, ColVII showed a pronounced increase in the hypoxic group at gene expression level, a finding that was confirmed at protein level (Fig. 1B and Supp. Table 2). To evaluate a potential role for ColVII in tumourigenesis we compared the estrogen-positive breast cancer cell line MCF-7 with the non-tumourigenic epithelial cell line MCF-12. First, we tested the proliferative activity by measuring Ki67 expression in each cell line. As expected, the MCF-7 cultures showed 30% more Ki67^+^ cells than the MCF-12 cultures (Fig. 1C). Immunofluorescence staining and western blotting showed that MCF-7 cultures had lower expression of ColVII compared with MCF-12 line (Fig. 1D-E). Furthermore Cyclin-D1 protein levels were lower in the MCF-12 cultures compared with the MCF-7 cultures (Fig. 1E).

### Increased collagen type VII expression correlates with lower MCF-7 proliferation rate

To evaluate the proliferative and invasion capabilities of MCF-7 cells in a ColVII rich environment in vitro, the cells were exposed to different concentration of human recombinant ColVII (rhColVII) (Fig. 2A). The GFP signal increased significantly until day ten in all the groups. However, MCF-7-GFP growing in plates coated with the highest rhColVII concentration showed lower GFP fluorescent signal (Fig. 2A), area occupied by GFP (Fig. 2B) at day 10 and reduction in Ki67^+^ cells after day 2 of culture (Fig. 2C). The lower proliferative capabilities of these MCF-7 cultures were consistent with elevated *P21* gene expression and *COL17A1*, a marker of low proliferative potential in breast cancer cell lines [15] (data not shown). There were no differences in MCF-7 invasiveness after 24 h exposure to different concentrations of rhColVII (data not shown).

### Collagen type VII is highly expressed by breast tissue stroma/mesenchymal cells

**Fig. 3:**
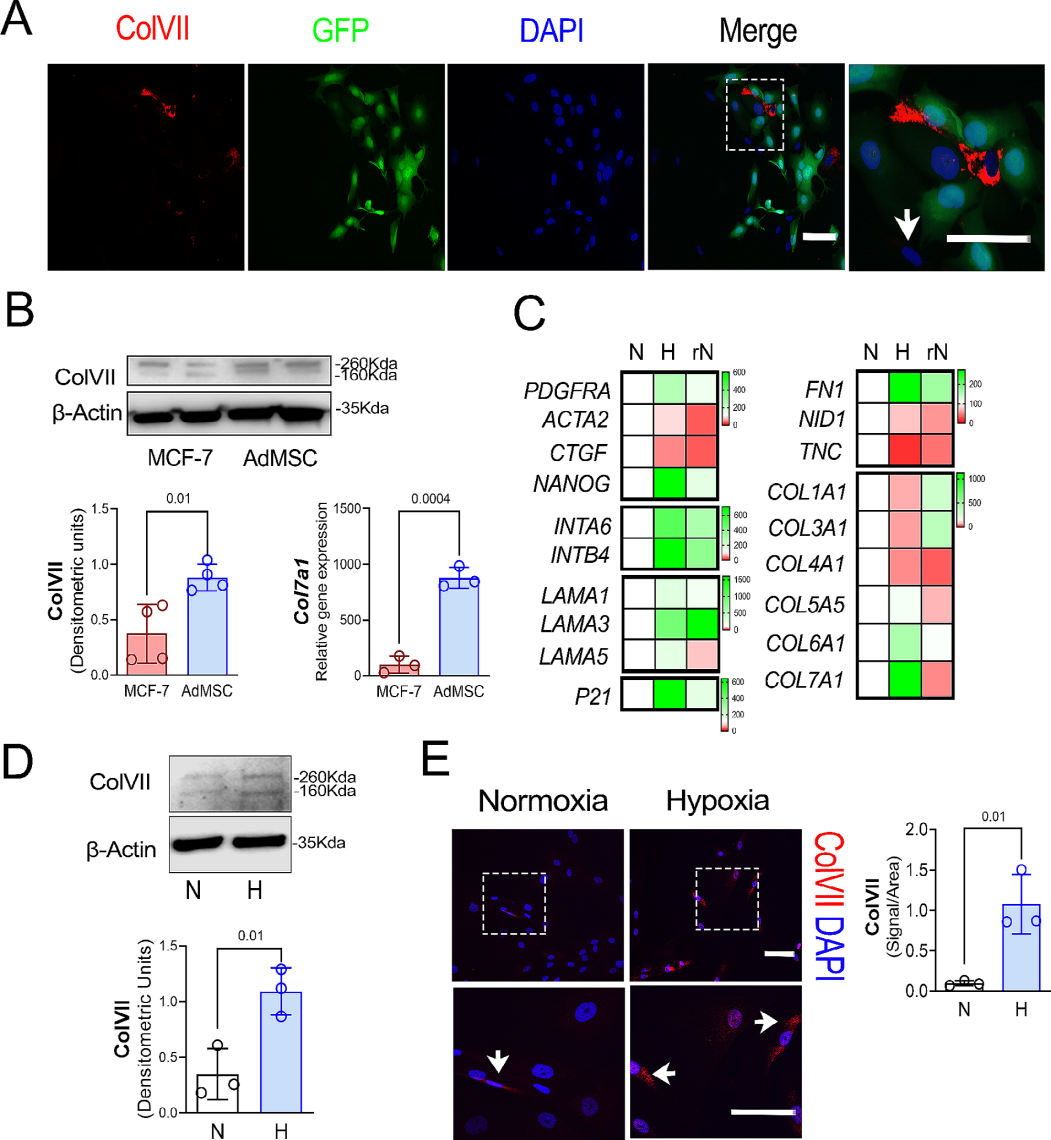
Collagen type VII is highly expressed by adipose tissue mesenchymal cells. **A.** Representative immunocytochemical staining of ColVII (Red) and GFP (Green) in MCF-7 and AdMSCs co-cultures. Nuclei are stained with DAPI (blue). Scale bar = 50 μm. Insets: Dotted areas´ magnifications. Arrow: non-Collagen type VII stained AdMSC. **B.** Above: Representative western blot against Collagen Type VII (ColVII) and β-Actin in MCF-7 and AdMSCs cultured for 5 days. Below: Quantification of Western blot bands (Densitometric units) showing relative expression of the target proteins to β-Actin. Data are expressed as means ± SEM of four experiments. *P*-values represent the t-tests between MCF-7 and AdMSCs. **C.** Gene expression analysis of fibroblast markers; *PDGFRα*, *ACTA2*,* CTGF*, stemness; *NANOG*, laminins; *LAMA1*, *LAMA3*, *LAMA5*, non-collagens; Fibronectin (*FN1*), Nidogen-1 (*NID-1*), Tenascin-C (*TNC*), Integrins; *INTα6* and INTβ4 and collagens; *COL1A1*, *COL3A1*, *COL4A1*, *COL5A1*, *COL6A1* and *COL7A1* in AdMSCs exposed to normoxia (N), hypoxia (H) and restored normoxia (rN). Data are expressed as means of triplicates and represents fold change relative to N (100) with colour coding (White = 100, Green > 100 and Red < 100). **D.** Above: Representative image of western blot against ColVII, and β-Actin in AdMSCs exposed to normoxia (N) and Hypoxia (H). Below: Quantification of Western blot bands (Densitometric units) showing relative expression of the target proteins to β-Actin Data are expressed as means ± SEM of triplicates. *P*-values represent the t-tests between N and H and H and rN. **E.** Left: Representative ColVII (Red) staining of AdMSCs exposed to normoxia and hypoxia. Nuclei are stained with DAPI (blue). Scale bar = 50 μm. Insets: Dotted areas´ magnifications. Right: Expression of positive ColVII signal quantification (Pixels) of AdMSCs exposed to normoxia (N) and hypoxia (H) per total cell area in the capture. Data are expressed as means ± SEM of triplicates. *P*-values represent the t-tests between N and H

Previous single cell transcriptome analyses have indicated that ColVII mainly is expressed by mesenchymal and fibroblasts-like cells rather than ductal epithelial cells in the breast tissue [22]. In line with Karlsson et al., we found that AdMSCs had a pronounced higher expression of ColVII at gene (data not shown) and protein levels (Fig. 3A, B) compared to MCF-7 cells. However, a marked difference in ColVII signal intensity was observed in the AdMSCs (Fig. 3A), suggesting different subpopulations of ColVII-expressing cells. To better understand how the hypoxic tumour microenvironment might affect mesenchymal stem cells, AdMSCs were treated with CoCl_2_ as previously described to analyse the gene response to hypoxia. The stem cell marker *NANOG* and undifferentiated stromal fibroblast marker *PDFGRα* were upregulated while, differentiated fibroblasts markers such as *ACTA2* and *CTGF* were significantly reduced under hypoxic conditions compared to the controls (Fig. 3C and Supp. Table 3). The hypoxic environments elevated ColVII expression at protein level (Fig. 3D, E) along with a reduction of fibroblast markers such as α-SMA (Supp. Figure 3A) or nidogen-1 (NID-1) (Supp. Figure 3B) [23].

### Integrin-α6 correlates with high ColVII expression

**Fig. 4:**
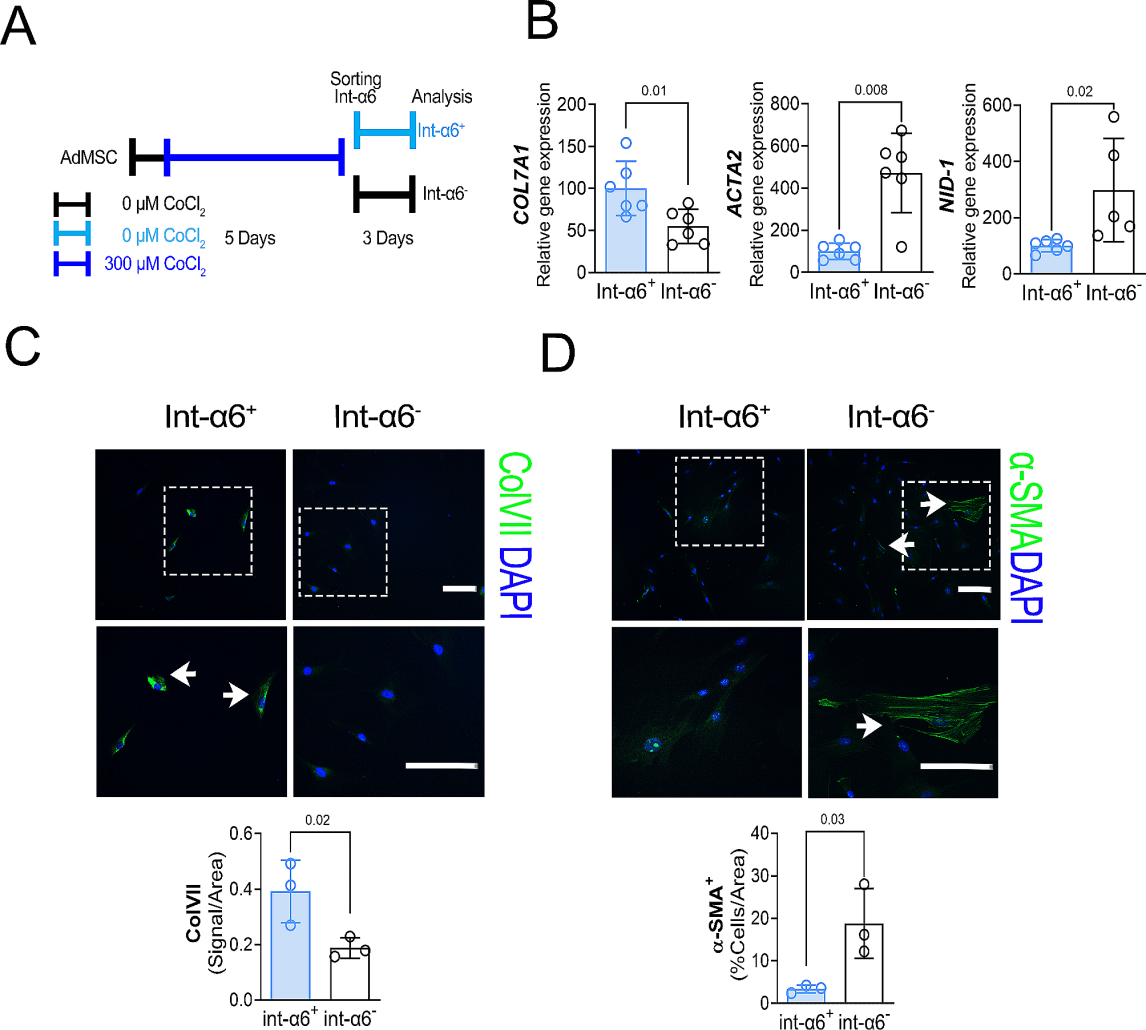
Integrin-α6 expression correlates with high Collagen type VII expression. **A.** Schematic picture of the experimental procedure to sort high and low ColVII expressing AdMSCs using cobalt chloride induced hypoxia and magnetic beads via anti Integrin-α6 antibody conjugated with APC. **B.** Gene expression analysis of *COL7A1*,* ACTA2* and *NID-1* in Integrin-α6^+^ (Int-α6^+^) and Integrin-α6^−^ (Intα-6^−^). Data are expressed as means ± SEM of 2 independent experiments with three wells in each. *P*-values were calculated by t-tests between Int-α6^+^vs. Int-α6^−^. **C.** Above: Representative immunocytochemical ColVII (green) staining of Integrin-6α^+^ (Int-α6^+^) and Integrin-6α^−^ (Intα-6^−^) populations. Nuclei are stained with DAPI (blue). Scale bar = 50 μm. Insets are magnifications of the selected dotted areas. Below: ColVII signal quantification (Pixels) per area of Integrin-6α^+^ (Int-α6^+^) and Integrin-6α^−^ (Intα-6^−^). Data are expressed as means ± SEM of triplicates. *P*-values were calculated by t-tests between Int-α6^+^vs. Int-α6^−^. **D.** Above: Representative immunocytochemical staining of α-SMA (green) staining. Nuclei are stained with Dapi (blue). Scale bar = 50 μm. Insets are magnifications of the selected dotted areas. Below: α-SMA signal quantification (Pixels) per area of Integrin-6α^+^ (Int-α6^+^) and Integrin-6α^−^ (Intα-6^−^). Data are expressed as means ± SEM of triplicates. *P*-values were calculated by t-tests between Int-α6^+^vs. Int-α6^−^

A subpopulation of AdMSCs expressing high levels of ColVII were isolated immunomagnetically using integrin-α6 specific antibodies (Fig. 4A). The Integrin-α6^+^ AdMSCs expressed higher ColVII (Int-α6^+^/ColVII_High_) along with lower myofibroblast markers such as *NID-1* or α-SMA at gene (Fig. 4B) and protein level (Fig. 4C-D) when compared with the integrin-α6^−^/ColVII_Low_ AdMSC population. Int-α6^+^/ColVII_High_ and int-α6^−^/ColVII_Low_ AdMSCs subpopulations were exposed to conditioned medium from MCF-7 cells and then compared with control medium. Conditioned medium from MCF-7 breast cancer cells induced higher cell migration in both subpopulations. However, significantly less Int-α6^+^/ColVII_High_ cells migrated when were compared with the Int-α6^−^ cells/ColVII_Low_ (Supp. Figure 4).

### Integrin-α6^+^/ColVII_High_ AdMSCs reduce proliferation of MCF-7 cells

**Fig. 5:**
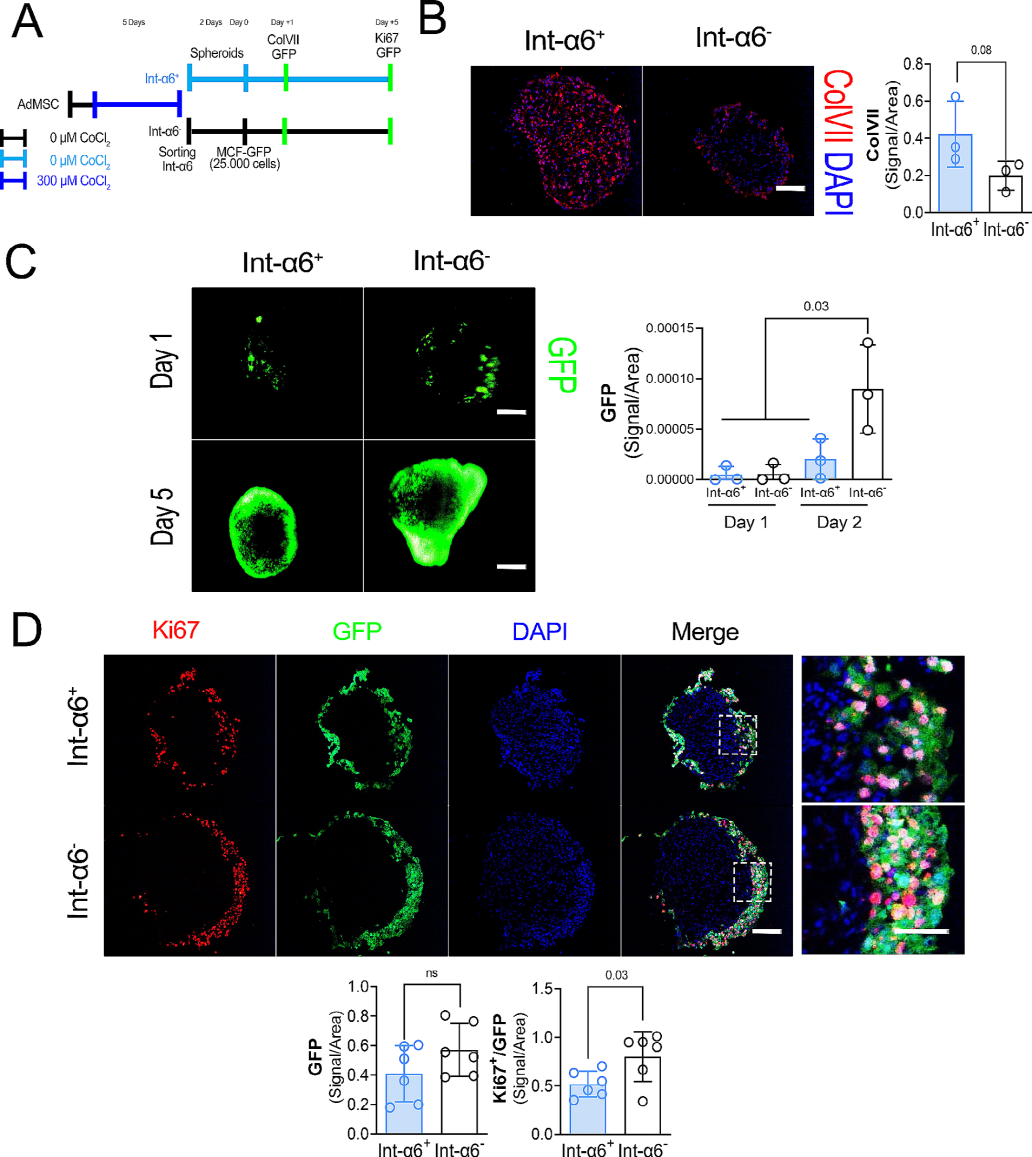
Spheroids of Collagen type VII high expressing AdMSCs reduce the proliferation of MCF-7 cells. **A.** Schematic picture of the experimental procedure to sort high and low ColVII expressing AdMSCs using CoCl_2_ induced hypoxia and magnetic beads via anti Integrin-α6 antibody conjugated with APC. Both subpopulations were used to generate spheroids. After one day, 25 000 MCF-GFP cells were put in contact with the spheroid’s cultures for 24 h. MCF-7 attached to AdMSC cocultures were transferred into a new vial, the GFP signal measured and cultured for 5 days. The GFP signal was measured at day 1 and day 5 and the expression of ki67 measured at day 5. **B.** Left: Representative immunocytochemical staining of ColVII (red) in Integrin-6α^+^ (Int-α6^+^) and Integrin-6α^−^ (Intα-6^−^) spheroids after 2–3 days of culture following sorting. Nuclei are stained with DAPI (blue). Scale bar = 50 μm. Right: ColVII signal quantification (Pixels) per area of Int-α6^+^ and Int-α6^−^ spheroids 2–3 days of spheroid culture. Data are expressed as means ± SEM of triplicates. *P*-values were calculated by t-tests between Int-α6^+^vs. Int-α6^−^. **C.** Left: Representative immunocytochemical staining of GFP in Int-α6^+^ and Int -α6^−^ spheroids after one and five days of coculture with MCF-7-GFP. Scale bar = 50 μm. Right: GFP signal quantification at day one and day five. Data are expressed as means ± SEM of triplicates. *P*-values were calculated by t-tests between Int-α6^+^vs. Int-α6^−^. **D.** Above: Representative immunocytochemical staining of Ki67 (red) and GFP (green) in Int-α6 + and Int-α6^−^ spheroids after 5 days of coculture with MCF-7-GFP. Scale bar = 50 μm. Below: GFP and Ki67 signal quantification on day five. Data are expressed as means ± SEM of three independent experiments with different number of spheroids combined, with each data point representing one spheroid. Means have been grouped and *p* value has been calculated by t-test

Three-dimensional AdMSCs-MCF-7 spheroids were generated to model solid tumour conditions (Fig. 5A). Integrin-α6^+^ AdMSC spheroids expressed two times more ColVII in comparison with integrin-α6^−^ AdMSC spheroids (Fig. 5B). Next MCF-7-GFP were added to the AdMSCs spheroids. Compared to Int-α6^−^/Col-VII_Low_ AdMSC spheroids, the Int-α6^+^/ColVII_High_ showed lower GFP signal 5 days after the inoculation of MCF-7 cells (Fig. 5C). Furthermore, MCF-7-GFP cells growing around Int-α6^+^/ColVII_High_ AdMSC spheroids showed a reduction in *Ki67* expression (GFP^+^Ki67^+^ population) compared with the Int-α6^−^/ColVII_Low_ AdMSC spheroids (Fig. 5D).

### Expression of ColVII in human breast cancer tissue

**Fig. 6:**
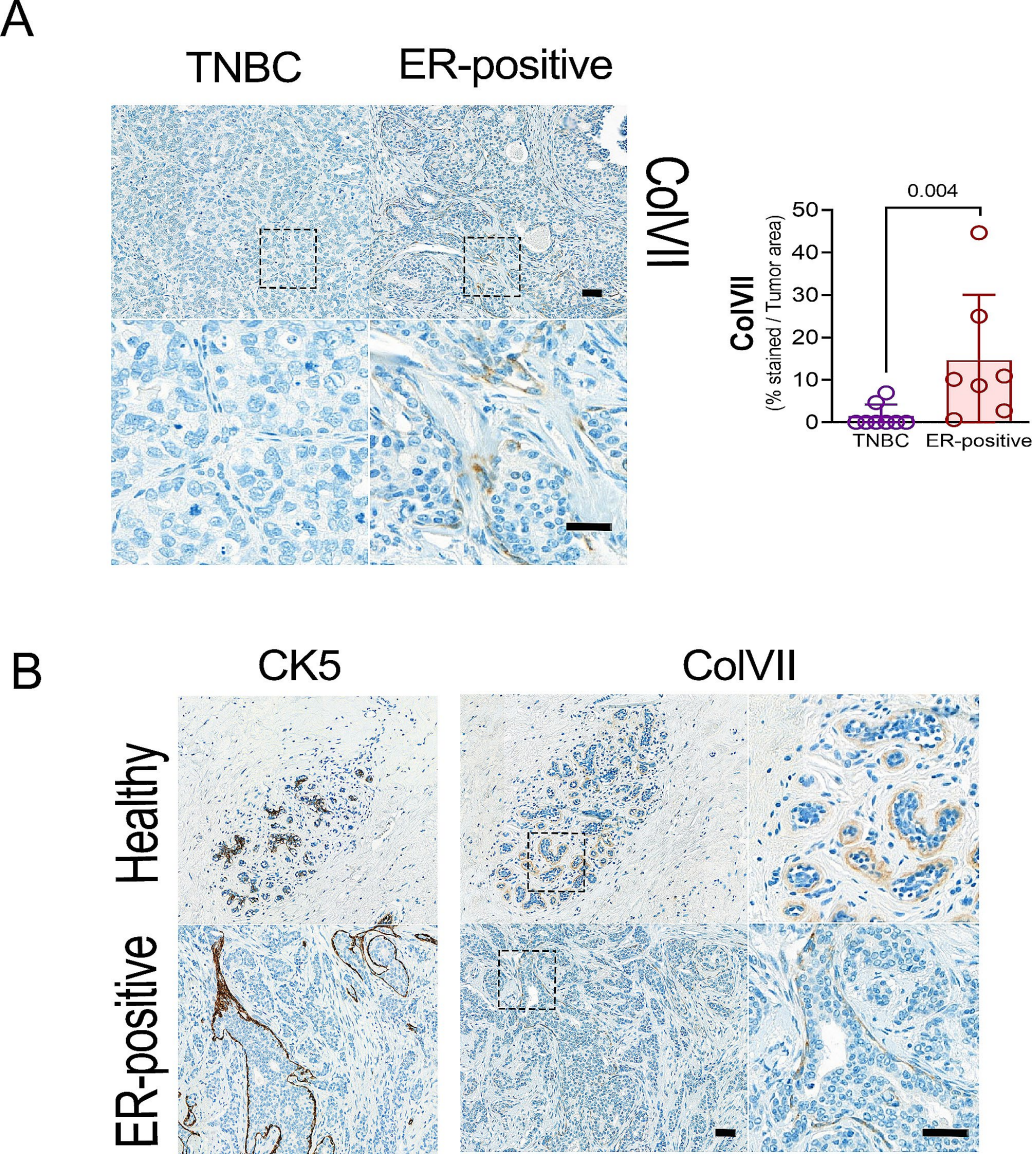
Collagen type VII expression in human breast tissue tumour area **A.** Left: Representative immunohistochemical ColVII staining in human triple negative breast cancer (TNBC) and estrogen receptor (ER) positive breast cancer. Scale bar = 100 μm. Right: Graph showing each patient’s (*n* = 15) percentage of positive ColVII-expression in tumour area stratified by hormone receptor status, either as ER-positive or TNBC. *P*-value was calculated by Mann-Whitney U test between ER-positive and TNBC. **B.** Above left: Representative immunohistochemical staining of CK5 in healthy breast terminal duct lobular unit of the mammary gland and ER-positive tumours. Above center: same histological site as above left showing representative ColVII staining and zoom in the selected area in right. Below left: Representative histological staining of CK5 in ER-positive breast tissue ducts. Below center: same histological site that below left showing a representative ColVII staining and zoom in the selected area in right. Scale bar = 100 μm

The expression of ColVII in the tumour area was analysed in samples from 15 patients and correlated to other known prognostic biomarkers. There was a significantly higher amount of ColVII expression in patients with low expression of the proliferation marker Ki67, in comparison with patients with high Ki67 expression (6/6 patients vs. 3/9 patients; *p* = 0,028). Similarly, higher ColVII expression was found in patients with nuclear grade 2 or less in comparison to patients with nuclear grade 3 (7/7 vs. 2/8; *p* = 0.007) as well as ER-positive breast cancer in comparison with TNBC (7/7 vs. 2/8) (Fig. 6A). There was however no significant difference of ColVII expression between the nodal positive vs. negative groups (4/8 vs. 5/7; *p* = 0.608). Histologically, a strong ColVII staining was found surrounding the mammary gland. Cytokeratin-5 staining indicated that the ColVII expressing cells were myoepithelial cells. Such staining was detected in the cancer area as well as in healthy surrounding stroma (Fig. 6B).

## Discussion

The present study described a potential role of ColVII in breast cancer cell proliferation. Using an in vitro model of hypoxia-induced dormancy, MCF-7 treated with CoCl2 to mimic hypoxia showed a significant increase in ColVII expression when compared with those maintained in normoxic conditions. Dormancy is a cell latent, non-proliferative cellular state. The expression of ColVII was associated with higher expression of the cycle arrest marker *P21* and other well-described ECM components impairing breast cancer growth.

In line with our in vitro results, we demonstrated that increased protein expression of ColVII in human breast cancer tissue correlated with well-known positive prognostic markers including low Ki67-expression, low tumour grade, and hormonal receptor positivity. Furthermore, we observed that the low proliferative breast epithelial cell line MCF-12 expressed a higher amount of ColVII along with lower Ki67 expression when compared to the more proliferative MCF-7 breast cancer cell line.

ColVII is an anchoring protein, important for the stability of the basement membrane through its non-collagenous 1 (NC1) domain that can bind to basement proteins with high affinity [24]. ColVII is a component of the hemidesmosomes´ anchoring fibrils, key structures for cell-to-ECM stability through the integrin α6β4 heterodimer receptors through laminin 332 [25, 26]. An altered distribution of integrin α6β4 heterodimers has been observed in primary breast carcinomas compared with benign breast lesions [27]. Along with an increased gene expression of ColVII under mimicked hypoxia, an increased expression of other basement membrane proteins like laminins, integrin α6β4 and COL4A1 was also observed in the AdMSC, suggesting a role of hypoxia in the base membrane niche homeostasis. ColVII is more expressed by stromal cells compared to ductal epithelial cells in the breast [28]. In the same manner, our AdMSCs presented higher expression of ColVII at gene and protein levels in comparison with MCF-7 cells. Interestingly, a marked variation in ColVII signal intensity was shown amongst the AdMSCs, suggesting different subpopulations for the ColVII expressing cells. In consequence, AdMSCs were sorted into high and low ColVII-expressing subpopulations, and were co-cultured with MCF-7 cancer cells in a 3D model of solid cancer to study their role in tumour growth. This resulted in a reduced proliferation of MCF-7 cells in the ColVII-enriched 3D model of solid cancer, pinpointing a different role for the high and low ColVII-expressing subpopulations of mesenchymal stem cells on breast cancer growth.

The ColVII-expressing cells surrounding the ducts in the tumour area appeared to be myoepithelial cells, which was confirmed with CK5 staining. The myoepithelial cells act as gatekeepers together with the basement membrane, preventing cancer cells from invading the surrounding stroma [29], maybe due to the role of ColVII hindering breast cancer proliferation as we observed in MCF-7 cultures growing on recombinant ColVII in vitro. Altered expression profiles and properties of myoepithelial cells have been implicated in progression to invasive carcinoma [30]. Taken together the observed correlation between ColVII and positive prognostic markers in human tissue, with the reduced proliferation of MCF-7 cells reported in the ColVII rich environment, we hypothesize that increased expression of ColVII by the surrounding myoepithelial cells might contribute to mammary gland duct basement membrane stability and hence preventing breast cancer cells progression.

The role of ColVII in carcinogenesis has been extensively studied in recessive dystrophic epidermolysis bullosa patients, as there is an increased risk of squamous cell carcinoma (SCC) at an early age in patients with ColVII deficiency [31]. Furthermore, siRNA targeting *ColVII* mediates increased SCC migration and invasion in vitro [32]. ColVII expression is both a positive and negative prognostic marker in other cancer types [15, 33, 34]. The incidence of advanced stage cancer and lymphatic invasion was shown to be significantly higher in the high ColVII expression group compared with the low ColVII-expression group in esophageal squamous cell carcinoma [34]. Furthermore, there is a significant association between ColVII immunohistochemistry score and distant metastasis in gastric cancer [33]. Our results are however consistent with a study performed by Hong et al., demonstrating that downregulation of COL7A1 was associated with a decreased 5-years survival rate in breast cancer [15].

In this present study we have assessed the role of ColVII in the breast cancer line MCF-7. However, the ColVII_High_ AdMSC were isolated indirectly using the hemidesmosome receptor Integrin α6, which makes it difficult to investigate the role of only ColVII. Future studies targeting ColVII and investigating mechanistically the role of ColVII in different cell types and signaling pathways must be conducted to better understand the potential role of ColVII affecting proliferation of breast cancer cells.

In conclusion, we hypothesize that ColVII is a positive prognostic marker in breast cancer, being associated with reduced proliferation of the breast cancer cell line MCF-7 in vitro as well as with several positive prognostic markers in human breast cancer tissue.

### Electronic supplementary material

Below is the link to the electronic supplementary material.

**Supplementary Material 1**: **Supplemental Fig. 1**. Collagen type VII antibody testing. Left; Representative images of ColVII staining in skin (positive control). Right: Representative staining of ColVII in liver (negative control).

**Supplementary Material 2**: **Supplemental Fig. 2**. Hypoxia induced dormancy in MCF-7 cells. A. Schematic overview of the experimental procedure to induce hypoxia in MCF-7 cells. MCF7-cells at a density of 5000 cells/cm^2^ were plated and allowed to settle for 2 days. After 2 days 300 µM cobalt chloride (CoCl_2_) was added to the hypoxic (H) and restored hypoxic (rH) groups. After 5 days the CoCl_2_ was removed in the rN group and new CoCl_2_ was added in the H group. B. RNA content (ng/µl) of MCF-7 cells exposed to normoxia (N), hypoxia (H) or restored normoxia (rN). Graphs show mean ± SEM. Data are expressed as means ± SEM of triplicates. *P*-values were calculated by t-tests between N vs. H and H. C. Left: Representative immunocytochemical staining of MCF-7-GFP cells exposed to normoxia, hypoxia or restored normoxia. Scale bar = 200 μm. Right: Relative GFP signal quantification in MCF-7-GFP exposed to normoxia (N), hypoxia (H) or restored normoxia (rN). Data are expressed as means ± SEM of triplicates. *P*-values were calculated by t-tests between N vs. H and N vs. rN. D. Gene expression analysis for *Ki67*,* P21*, *NANOG* and *OCT3*/4 in MCF-7 exposed to normoxia (N), hypoxia (H) or restored normoxia (rN). Data are expressed as means ± SEM of 2 independent experiments and are presented as fold change relative to H. *P*-values were calculated by one way ANOVA.

**Supplementary Material 3**: **Supplemental Fig. 3**. Hypoxia reduces fibrogenesis in AdMSCs. A. Left: Representative immunocytochemical staining of α-SMA (green) in AdMSCs in normoxia and hypoxia. Nuclei are stained with DAPI (blue). Scale bar = 50 μm. Insets are magnifications of the selected dotted areas. Right: α-SMA expression quantification in AdMSCs exposed to normoxia (N) and hypoxia (H) for 5 days. Data are expressed as means ± SEM of triplicates. *P*-values were calculated by t-tests between N vs. H. B. Representative immunocytochemical staining of Nidogen-1 (NID-1) (red) in AdMSCs in normoxia and hypoxia. Nuclei are stained with DAPI (blue). Scale bar = 50 μm. Insets are magnifications of the selected dotted areas. Right: NID-1 expression quantification in AdMSCs exposed to normoxia (N) and hypoxia (H) for 5 days. Data are expressed as means ± SEM of triplicates. *P*-values were calculated by t-tests between N vs. H.

**Supplementary Material 4**: **Supplemental Fig. 4**. MCF-7 secretome has lower impact recruiting Int-α6^+^ AdMSCs. Integrin-6α^+^ (Int-α6^+^) and Integrin-6α^−^ (Intα-6^−^) AdMSCs migration values (480/582nm) after 24 h exposed to MCF-7 or Control (MCF-7 secretome free) conditioned media. Data are expressed as means ± SEM of four experiments. Graphs are represented as mean ± SEM. Each data point is the mean value of *n* = 4 independent experiments grouped. *P*-values have been calculated by one way ANOVA.

**Supplementary material 5**

**Supplementary material 6**

**Supplementary material 7**

## Data Availability

No datasets were generated or analysed during the current study.
